# Safe discharge on the second postoperative day after major colorectal surgery: a decision-making strategy based on quantitative serological data

**DOI:** 10.1007/s10151-026-03300-0

**Published:** 2026-03-27

**Authors:** M. Kraft, B. van Doorn, I. Maya, A. Solís-Peña, G. Pellino, E. Espín-Basany

**Affiliations:** 1 Department of Surgery and Morphological Sciences, Universitat Autònoma de Barcelona UAB, Barcelona, Spain; 2 Colorectal Surgery, Department of General and Digestive Surgery, Vall d’Hebron University Hospital, Barcelona, Spain

**Keywords:** CRP, ERAS, C-reactive protein, Second postoperative day, Anastomotic leak, Colorectal surgery, Complications

## Abstract

**Background:**

Enhanced Recovery After Surgery (ERAS) has enabled early patient discharge, but reliable biomarkers are needed to support safe early discharges. C-reactive protein levels have been reported as good markers for early anastomotic leak detection and also for overall complications, but there is no consensus yet on its quantification nor day of analysis.

**Objective:**

This study aimed to determine the C-reactive protein cutoff values at postoperative day (POD) 2–4 associated with the lowest risk of postoperative complications.

**Design:**

Single-center, retrospective study.

**Settings:**

Tertiary hospital.

**Patients:**

Patients operated on between 2019 and 2022.

**Interventions:**

Surgery for colorectal cancer.

**Main outcome measures:**

C-reactive protein on postoperative days 2–4 and the delta difference between postoperative days 3 and 2 were measured, identifying the best cutoff value for each postoperative complication. Receiver-operating characteristics curves were generated and the area under the curve was analyzed for each postoperative day measurement.

**Results:**

A total of 434 patients were included, median age was 72 (62–80) years. On postoperative day-2, the cutoff values for overall morbidity, surgical complications, medical complications, and anastomotic leak were 139.2 mg/L, 144.4 mg/L, 140 mg/L, and 170 mg/L, respectively. POD3 and 2 were both safe (area under the curve of 0.7507 and 0.7466, respectively). The negative predictive values using a C-reactive protein POD2 cutoff value of 140 mg/L were 80.6%, 91.5%, 87.6%, and 98.6% for global, medical, surgical complications, and anastomotic leak, respectively.

**Limitations:**

Retrospective study.

**Conclusions:**

Carefully selected, motivated, and clinically suitable patients could be offered discharge on POD2 if their C-reactive protein levels are below 140 mg/L, with a very high negative predictive value for anastomotic leak and other postoperative complications. This decision should be made in collaboration with patients, considering clinical assessment and logistic factors.

**Supplementary Information:**

The online version contains supplementary material available at 10.1007/s10151-026-03300-0.

## Introduction

Colorectal cancer (CRC) is the third most deadly malignancy worldwide, and its incidence is increasing each year [1, 2]. Most patients will need surgical resection and it is known that long-term survival can be impaired by short-term complications. Anastomotic leak (AL) is the most feared complication, and its early detection is crucial to lower postoperative mortality.

At a time when most of colorectal units around the world follow Enhanced Recovery After Surgery (ERAS) protocol in their everyday practice, allowing for early discharge of patients [3], there is still need of biological markers to support safe discharges for patients undergoing colorectal resection. In this regard, C-reactive protein (CRP) levels have been widely reported in the literature for early detection of AL [4–6], and several studies have suggested that it can be used to identify patients that are less likely to have developed a subclinical complication [7, 8] and might therefore be suitable for early discharge.

Even if length of stay (LOS) in the hospital might not be much relevant to most patients, identifying those who might be offered an early return home might help in speeding the postoperative recovery of patients, in a familiar environment, and might allow for the treatment of a higher number of patients with CRC in shorter time intervals, which is relevant in the post-pandemic era. Eventually, this might also help in reducing the costs for the health services. CRP can be used as an adjunct to clinical assessment to detect adverse events that have not yet produced signs or symptoms. However, there is no agreement on the actual relevance of CRP and no globally agreed quantification of CRP values that exists for this population of patients. CRP cutoff thresholds range widely across different studies, with values going from 92 to 200 mg/L at different postoperative days (POD) [9]. As an example, a recent study published by Gielen et al. [10] established cutoff values of 170 mg/L on POD2 and 152 mg/L on POD3 as a risk factor for major complications after colorectal surgery. Most of the studies analyze POD3 and POD4 and are focused on detecting major complications rather than detecting patients who are most likely not to present them.

This study aimed to determine the CRP cutoff values for POD2–4 that are associated with the lowest risk of postoperative complications to identify those values that can allow for a safe hospital discharge on POD2 in patients who are clinically suitable and wish to return home early.

## Methods

A single-center, observational and retrospective study was designed on the basis of a prospective database, including patients operated between December 2019 and January 2022 at Vall d’Hebron University Hospital (Barcelona, Spain). Ethical approval from the ethical committee was granted. The study was performed and is reported according to the Strengthening The Reporting of Observational Studies in Epidemiology (STROBE) statement [11].

### Eligibility criteria

All patients who underwent major elective surgery for neoplasms in the colon or rectum and received an anastomosis were evaluated for inclusion. Patients undergoing surgery for inflammatory bowel disease, diverticular disease, multivisceral resection, emergency surgery or transanal minimally invasive surgery were excluded from the analyses.

### Early detection program for AL

At the center where the procedures were performed, a specific protocol of early diagnosis of AL is followed. All patients are tested for CRP levels on POD3. If the value is higher than 140 mg/L, CRP and procalcitonin (PCT) determination is requested on POD4, and if CRP or PCT are higher than > 125 mg/L and > 0.41 ng/mL, respectively, or if CRP increases from POD3 to POD4, computed tomography (CT) scan is performed to rule out AL [12]. This applies to asymptomatic patients; if clinical symptoms are observed suggestive of AL, CT scan is performed in any CRP or PCT level. For this study, CRP determination was also performed on POD2.

### Variables of interest

Patient age, sex, American Society of Anesthesiologists (ASA) classification [13], and type of surgery were analyzed. Outcome variables included mortality, overall morbidity (all deviations from the normal postoperative course), surgical complications (including surgical site infections (SSI), postoperative ileus, anastomotic bleeding, colonic ischemia, occlusion and iatrogenic lesions), medical complications (including cardiac, gastrointestinal, hematologic, nephro-urinary, respiratory, neurologic, and vascular complications), AL, and surgical wound complications (including surgical wound infections, hematomas, and evisceration). AL was defined as clinical or radiological suspicion of AL, including intrabdominal collections. All postoperative complications were graded according to the Clavien–Dindo classification [14]. CRP was recorded on POD2 and POD3 for all patients, and CRP and PCT were recorded on POD4 if CRP POD3 was higher than > 125 mg/L.

### Endpoints and outcome measure

The primary endpoint was to identify the best value of CRP to be used for a safe POD2 discharge after colorectal surgery for cancer. The outcome was the best cutoff value for each different postoperative complication at POD2.

Secondary outcomes included the creation of areas under the curve (AUC) of each POD for global morbidity, as well as for the delta difference between the different postoperative days (POD4–2, POD4–3 and POD3–2). This was assessed using the correlation between each POD CRP and any postoperative complication, and it was stratified according to Clavien–Dindo grade. A subanalysis was also carried out, stratifying per age (above or under 70 years of age).

The sensitivity, specificity, positive predictive value (PPV), and negative predictive value (NPV) of POD2 CRP were used for the scope. A CRP value for POD2 was chosen to use as cutoff in our daily practice.

### Statistical methods

Receiver-operating characteristic (ROC) curves were generated for CRP values at each POD, as well as for PCT and the delta difference between POD3 and POD2, according to each type of postoperative complication. The best cutoff value was identified using the Youden criteria. The area under the ROC curve (AUC) was calculated to know which POD value was the most accurate. To evaluate the AUC, a standard academic point system was adopted: 0.90–1 = excellent, 0.80–0.90 = good, 0.70–0.80 = fair, 0.60–0.70 = poor, and 0.50–0.60 = fail. Qualitative variables are presented as absolute values and percentages (%), while continuous variables are presented as medians with 25th and 75th percentiles and interquartile ranges (IQR). The Student’s *t*-test was used for qualitative variables comparisons. *P*-values below 0.05 were considered to be statistically significant.

The statistical analysis was performed using Stata 15.1.

### Ethical considerations

The study was approved by the local Ethical Committee.

## Results

**Fig. 1:**
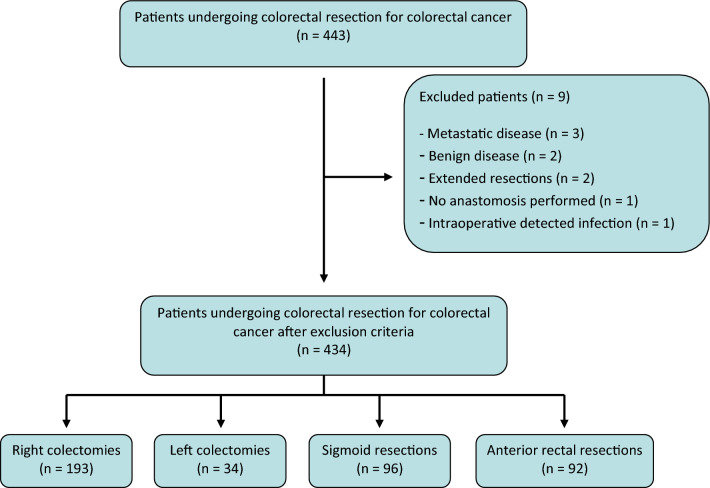
Patient flowchart

In total, 443 patients met the inclusion criteria. After revising medical histories, 9 patients were excluded from the final analysis, in which 434 patients were included (Fig. 1). Procedures breakdown was as follows: 193 right colectomies (44.5%), 34 left colectomies (7.8%), 96 sigmoid resections (22.1%), and 92 anterior rectal resections (21.2%). The median age of the patients was 72 (62–80) years, with 253 (58%) male patients. The median CRP value was 114 mg/L (IQR 94), 109.4 mg/L (IQR 113.2), and 150 mg/L (IQR 118.3) for POD2, POD3, and POD4, respectively, and the median PCT on POD4 was 0.38 ng/mL (IQR 0.93) (Table 1).

**Table 1:** Baseline characteristics

Variable	*N* = 434
Age, years	72 (62–80)
Gender, male	253 (58)
ASA classification	
ASA I	56 (12.9)
ASA II	235 (54.2)
ASA III	137 (31.6)
ASA IV	6 (1.4)
Procedure performed	
Right colectomy	193 (44.5)
Left colectomy	34 (7.8)
Sigmoidectomy	96 (22.1)
Anterior rectal resection	92 (21.2)
CRP POD2	114 mg/L (73–167)*
CRP POD3	109.4 mg/L (60.2–173.4)^†^
CRP POD4	150.0 mg/L (103–221.3) (136)^‡^
PCT POD4	0.38 ng/mL (0.2–1.13) (97)^§^

Categorical values are reported as absolute numbers (%); continuous values are reported as median (25th–75th percentile)

*ASA* American Society of Anesthesiologists, *CRP* C-reactive protein, *POD* postoperative day, *PCT* procalcitonin

*n = 417

^†^*n* = 387

^‡^*n* = 136

^§^*n* = 97

The overall morbidity rate, including both medical and surgical complications, was 32%, with the rate of surgical complications at 22.8% and the rate of medical complications at 15.5%. A total of 30 patients (6.9%) were diagnosed with an AL, and 37 (8.6%) required reoperation. SSI occurred in 13.1%. The mortality rate was 3.2% (Table 2).

**Table 2:** Postoperative complications

	*N* = 434
Overall morbidity	139 (32)
Surgical complications	99 (22.8)
Anastomotic leakage	30 (6.9)
Reintervention	37 (8.6)
Surgical site infection	57 (13.1)
Superficial	6 (1.4)
Organ-space	51 (11.7)
Surgical wound complications	11 (2.5)
Medical complications	67 (15.5)
Overall mortality	14 (3.2)
Clavien–Dindo	139 (100)
Clavien–Dindo I	10 (7.2)
Clavien–Dindo II	84 (60.4)
Clavien–Dindo IIIa	1 (0.7)
Clavien–Dindo IIIb	22 (15.9)
Clavien–Dindo IV	8 (5.8)
Clavien–Dindo V	14 (10.1)

Values are reported as absolute numbers (%)

### Primary outcome

On POD2, the best CRP cutoff value for overall morbidity was 139.2 mg/L. The best cutoff value for surgical complications was 144.4 mg/L, 140 mg/L for medical complications, and 170 mg/L for AL. Sensitivity, specificity, PPV, and NPV are described in Table 3.

**Table 3:** Cutoff values of C-reactive protein for different adverse events on postoperative day 2

Variable	Ideal CRP cutoff value (mg/L)	Sensitivity	Specificity	PPV	NPV
Global morbidity	139.2	65.9	74.7	53.8	83
Medical complications	140	70.9	68.9	28.6	93.1
Surgical complications	144	66.7	75.1	44.4	88.3
AL	170	76.7	79.6	22.6	97.8

Sensitivity, specificity, positive predictive value, and negative predictive value are reported as percentages

*CRP* C-reactive protein, *PPV* positive predictive value, *NPV* negative predictive value, *AL* anastomotic leakage

### Secondary outcomes

**Fig. 2:**
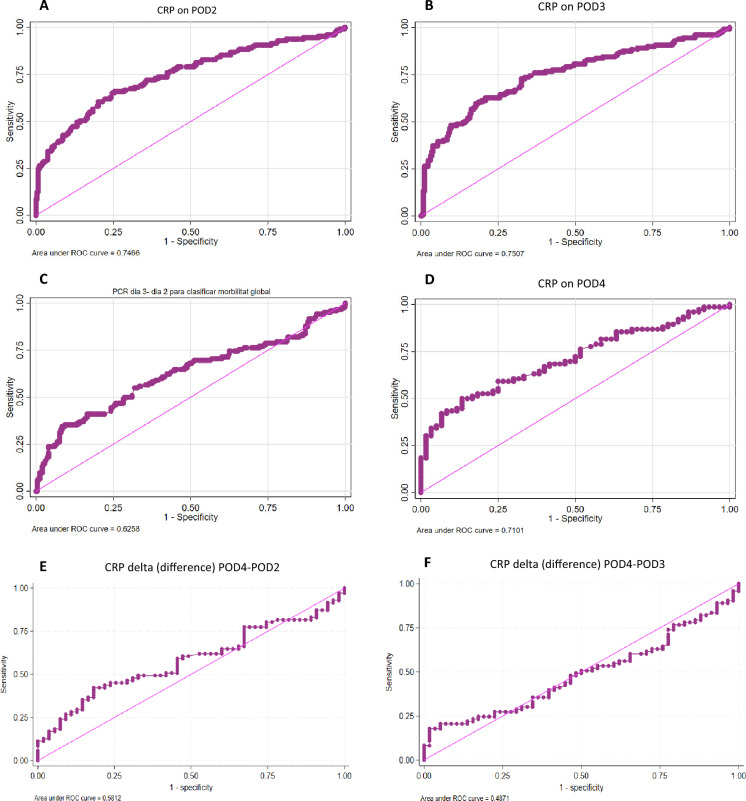
**A** Receiver-operative characteristics (ROC) curve for C-reactive protein (CRP) on postoperative day 2 (POD2). **B** Receiver-operative characteristics (ROC) curve for C-reactive protein (CRP) on POD3. **C** Receiver-operative characteristics (ROC) curve for C-reactive protein (CRP), delta (Δ, difference) POD3–2 values. **D** Receiver-operative characteristics (ROC) curve for C-reactive protein (CRP) on POD4. **E** Receiver-operative characteristics (ROC) curve for C-reactive protein (CRP), delta (Δ, difference) POD4–2 values. **F** Receiver-operative characteristics (ROC) curve for C-reactive protein (CRP), delta (Δ, difference) POD4–3 values

The ROC curve AUC for overall morbidity was assessed from POD2 to POD4. The highest AUC was found for CRP on POD3 with a value of 153 mg/L (AUC 0.7507), followed by CRP on POD2 with a value of 139.2 mg/L (AUC 0.7466). AUC for the delta differences between CRP on POD3 and POD2, on POD4 and POD2 and on POD4 and POD3, and AUC for CRP on POD4 was lower (AUC 0.6358, AUC 0.5812, AUC 0.4871, and AUC 0.7101, respectively). Table 4 summarizes the findings. The ROC curves can be seen in Fig. 2A–F.

**Table 4:** Ideal cutoff point for global morbidity

CRP values at different intervals	Cutoff point (mg/L)	AUC
CRP on POD2	139.2	0.7466
CRP on POD3	153	0.7507
CRP on POD4	193	0.71
CRP delta (difference) POD3–2	28	0.6258
CRP delta (difference) POD4–2	13	0.5812
CRP delta (difference) POD4–3	21	0.4871

*POD* postoperative day, *CRP* C-reactive protein, *AUC* area under the curve

The subanalysis stratifying patients by age showed slight differences on POD2, with CRP values of 176 mg/L (AUC 0.7621) for patients under 70 years compared with CRP values of 140 mg/L (AUC 0.7327) for patients older than 70 years, while values on POD3 were more similar, with CRP values of 150 mg/L (AUC 0.7586) for patients younger than 70 years compared with CRP values of 142 mg/L (AUC 0.7394) for patients older than 70 years (Supplement—Table 2, Supplement—Fig. 1).

CRP values on POD2 were higher in patients having postoperative global morbidity, mortality, and patients who needed reoperation (*p* < 0.0001) (Supplement—Table 1).

Using a POD2 CRP value of 140 mg/L as cutoff, the NPV for global morbidity, medical complications, surgical complications, and AL was 80.6, 91.5, 87.6, and 98.6%, respectively (Table 5).

**Table 5:** Screening tests for CRP cutoff value of 140 mg/L on POD2

Variable	Sensitivity	Specificity	PPV	NPV
Global morbidity	59.6	76.5	53.6	80.6
Medical complications	64.2	70.8	28.7	91.5
Surgical complications	64.6	74	42.4	87.6
AL	86.7	69.1	17.2	98.6

Sensitivity, specificity, positive predictive value, and negative predictive value are reported as percentages

*PPV* positive predictive value, *NPV* negative predictive value, *AL* anastomotic leakage

## Discussion

This study was able to identify the best POD2 CRP cutoff values for the most common complications after colorectal surgery, which were set to 139.2 mg/L for overall morbidity, 144.4 mg/L for surgical complications, 140 mg/L for medical complications, and 170 mg/L for AL. A CRP value of 140 mg/L was associated with a NPV near to 80% for global morbidity, around 90% for medical and surgical complications, and almost of 98% for AL. In a daily clinical practice, rather than proposing different thresholds for different types of complications, a single and easy applicable value is more useful for guiding clinical decision-making. It can therefore be suggested that a CRP value of 140 mg/L on POD2 is a safe cutoff to suspect a complication and to identify patients who might be offered early discharge. Of note, the safest CRP cutoff value according to AUC was found to be that of POD3 (0.7507), but the difference with the POD2 value was minimal (0.7466).

Previous studies have suggested a potential for CRP to predict postoperative complications. Welsch et al. found that a CRP above 140 mg/L on POD3–4 is predictive of infectious postoperative complications [15]. Ortega-Deballon et al. suggested a NPV of 95.8% for AL at POD4 with a cutoff value of 125 mg/L [16], with AUC for POD2 and POD4 CRP corresponding to 0.706 and 0.804, respectively. The small differences in values between the studies can be explained by several confounders, including the differences of AL rates, the definitions used for the different complications, and the differences in the chosen population. The delta differences between POD4, POD3, and POD2 were not a good marker of global morbidity, as they had the lowest AUC. This could be explained because values of inflammatory markers show their peaks at POD2 and then decrease in both patients with no complications as in patients with complications. Therefore, absolute values are more important than tendencies, which is consistent with our work [16, 17].

The findings of the current study should be interpreted with caution, though. Readers are to be advised that a CRP value below 140 mg/L on POD2 cannot be used per se to discharge all patients home on the second day after surgery. The decision to discharge patients should be agreed upon with the patients themselves and their relatives, and the benefits of an early return to their leisure and everyday activities should be weighed against the risk for a potential complication occurring while away from the hospital, which could go undiagnosed, delaying treatment and implying a physical and mental stress for the individual. The authors would suggest a very prudent and selective strategy, taking into account additional factors, such as patient independence and motivation, geographical factors (patients residing at an acceptably near place are to be preferred for the approach), capability of detecting signs and symptoms of alarm, and the availability of a 24-h on-call team that could admit the patients, should an adverse event be suspected.

Tavernier et al. recently suggested that considering four clinical criteria (return of bowel function, tolerance of diet, pain less than 5 out of 10 on a visual analogue scale, and being non-febrile during the entire stay) in addition to CRP levels below 150 mg/L on the day of discharge, increases the sensitivity for the diagnosis of AL from 60% to 86.7% and diminishes the false-negative rate from 40% to 13.3% [18]. Once again, this highlights the importance of assessing the patient clinically and to discuss shorter stay and the associated benefits and risks on an individual basis. Notwithstanding such a caveat, the importance of having a reliable and objective decision-making tool should not be underestimated.

### Study limitations and strengths

Our study has limitations. It is a single-center, retrospective study of patients operated on at a tertiary hospital. However, strengths of the study include the prospective data collection, the consistent use of the perioperative protocol for early detection of complications, and the high number of patients operated on in an adequately short timeframe to avoid confounders (e.g., changes in instrumentation, operating surgeons, and disease treatment) affecting the findings. In addition, all patients were consistently managed following the ERAS protocol, making the findings more easily generalizable and limiting the impact of perioperative variables. The fact that a reliable cutoff value of a routinely performed, inexpensive, and globally available biomarker such as CRP was identified offers a valuable adjunct in the decision-making for early discharge of those patients who are suitable for such a practice and are strongly motivated to return home on POD2.

## Conclusions

Carefully selected, motivated, and clinically suitable patients could be offered discharge on POD2 if CRP values are below 140 mg/L, with very high NPV for AL and complications. The decision must be made on the basis of clinical assessment and logistic factors for discharge, and must take into account the will of the patients and their relatives. It is desirable that the promising findings of the current report are confirmed in multicentric prospective studies.

## Supplementary Information

Below is the link to the electronic supplementary material.

Supplementary file1 (DOC 49 KB)

## Data Availability

No datasets were generated or analyzed during the current study.

## References

[1] Rawla P, Sunkara T, Barsouk A (2019) Epidemiology of colorectal cancer: incidence, mortality, survival, and risk factors. Gastroenterol Rev 14(2):89–103 10.5114/pg.2018.81072 PMC6791134 31616522

[2] Mármol I, Sánchez-de-Diego C, Pradilla Dieste A, Cerrada E, Rodriguez Yoldi MJ (2017) Colorectal carcinoma: a general overview and future perspectives in colorectal cancer. Int J Mol Sci 18(1):197 28106826 10.3390/ijms18010197 PMC5297828

[3] ERAS Compliance Group (2015) The impact of enhanced recovery protocol compliance on elective colorectal cancer resection: results from an international registry. Ann Surg 261(6):1153–1159 25671587 10.1097/SLA.0000000000001029

[4] Messias BA, Botelho RV, Saad SS, Mocchetti ER, Turke KC, Waisberg J (2020) Serum C-reactive protein is a useful marker to exclude anastomotic leakage after colorectal surgery. Sci Rep 10(1):1687 32015374 10.1038/s41598-020-58780-3 PMC6997159

[5] Platt JJ, Ramanathan ML, Crosbie RA et al. (2012) C-reactive protein as a predictor of postoperative infective complications after curative resection in patients with colorectal cancer. Ann Surg Oncol 19(13):4168–4177 22805866 10.1245/s10434-012-2498-9

[6] Garcia-Granero A, Frasson M, Flor-Lorente B et al. (2013) Procalcitonin and C-reactive protein as early predictors of anastomotic leak in colorectal surgery: a prospective observational study. Dis Colon Rectum 56(4):475–483 23478615 10.1097/DCR.0b013e31826ce825

[7] Gozalichvili D, Binquet C, Boisson C, Guiraud A, Facy O, Ortega-Deballon P (2023) Early detection of anastomotic leak with C-reactive protein increases the chances of anastomotic salvage. Colorectal Dis 25(4):728–737 36323646 10.1111/codi.16399

[8] Sala Hernandez A, Frasson M, García-Granero A et al. (2021) Diagnostic accuracy of C-reactive protein, procalcitonin and neutrophils for the early detection of anastomotic leakage after colorectal resection: a multicentric, prospective study. Colorectal Dis 23(10):2723–2730 34314565 10.1111/codi.15845

[9] Gans SL, Atema JJ, van Dieren S, Groot Koerkamp B, Boermeester MA (2015) Diagnostic value of C-reactive protein to rule out infectious complications after major abdominal surgery: a systematic review and meta-analysis. Int J Colorectal Dis 30(7):861–873. 10.1007/s00384-015-2205-y. (**Epub 2015 May 3. PMID: 25935447; PMCID: PMC4471323**) 25935447 10.1007/s00384-015-2205-y PMC4471323

[10] Gielen AHC, Schoenmakers M, Breukink SO et al. (2024) The value of C-reactive protein, leucocytes and vital signs in detecting major complications after oncological colorectal surgery. Langenbecks Arch Surg 409:76. 10.1007/s00423-024-03266-3 38409295 10.1007/s00423-024-03266-3 PMC10896856

[11] von Elm E, Altman DG, Egger M, Pocock SJ, Gøtzsche PC, Vandenbroucke JP (2007) STROBE Initiative. The Strengthening the Reporting of Observational Studies in Epidemiology (STROBE) statement: guidelines for reporting observational studies. Lancet 370(9596):1453–1457 18064739 10.1016/S0140-6736(07)61602-X

[12] Vallribera F, Kraft M, Pera M, Vidal L, Espín-Basany E (2021) Outcomes of intra- versus extra-corporeal ileocolic anastomosis after minimally invasive right colectomy for cancer: an observational study. J Clin Med 10(2):307 33467636 10.3390/jcm10020307 PMC7830629

[13] Hurwitz EE, Simon M, Vinta SR et al. (2017) Adding examples to the ASA-physical status classification improves correct assignment to patients. Anesthesiology 126(4):614–622 28212203 10.1097/ALN.0000000000001541

[14] Dindo D, Demartines N, Clavien PA (2004) Classification of surgical complications: a new proposal with evaluation in a cohort of 6336 patients and results of a survey. Ann Surg 240:205–213 15273542 10.1097/01.sla.0000133083.54934.ae PMC1360123

[15] Welsch T, Müller SA, Ulrich A et al. (2007) C-reactive protein as early predictor for infectious postoperative complications in rectal surgery. Int J Colorectal Dis 22(12):1499–1507 17639424 10.1007/s00384-007-0354-3

[16] Ortega-Deballon P, Radais F, Facy O et al. (2010) C-reactive protein is an early predictor of septic complications after elective colorectal surgery. World J Surg 34(4):808–814 20049435 10.1007/s00268-009-0367-x PMC2877195

[17] Facy O, Paquette B, Orry D et al. (2016) IMACORS study. Diagnostic accuracy of inflammatory markers as early predictors of infection after elective colorectal surgery: results from the IMACORS study. Ann Surg 263(5):961–966 26135691 10.1097/SLA.0000000000001303

[18] Tavernier C, Flaris AN, Passot G, Glehen O, Kepenekian V, Cotte E (2022) Assessing criteria for a safe early discharge after laparoscopic colorectal surgery. JAMA Surg 157(1):52–58 34730770 10.1001/jamasurg.2021.5551 PMC8567183

