# Supplementary material for: Safe discharge on the second postoperative day after major colorectal surgery: a decision-making strategy based on quantitative serological data
Source: Tech Coloproctol. 2026 Mar 27;30(1):48. doi: 10.1007/s10151-026-03300-0 (PMC13031230; doi:10.1007/s10151-026-03300-0)
Supplement: Supplementary file 1 — Supplementary file1 (DOC 49 KB) [file 10151_2026_3300_MOESM1_ESM.doc]

Supplementary Material:

| **Suppl. Table 1. Correlation of POD2 CRP values and postoperative outcomes** | | | |
| --- | --- | --- | --- |
| **Variable** | **Global morbidity** | | **p-value** |
|  | **No** | **Yes** |  |
| **CRP 2nd POD** | 9.8 (6.4 – 14) | 16.7 (10.9 – 24.5) | <0.0001 |
| **Variable** | **Mortality** | |  |
|  | **No** | **Yes** |  |
| **CRP 2nd POD** | 11.3 (7.2 – 16.4) | 21.7 (14.2 – 25.9) | <0.0001 |
| **Variable** | **Re-operation** | |  |
|  | **No** | **Yes** |  |
| **CRP 2nd POD** | 10.8 (7 – 15.5) | 19.5 (14.7 – 22.9) | <0.0001 |
| POD: post-operative day; CRP: C reactive protein | | | |

| **Suppl. Table 2. Ideal Cut-off point for global morbidity** | | | | |
| --- | --- | --- | --- | --- |
| **CRP values at different intervals** | **< 70 years** | | **> 70 years** | |
|  | **Cut-off point (mg/L)** | **AUC** | **Cut-off point (mg/L)** | **AUC** |
| **CRP on POD2** | 176 | 0.7621 | 140 | 0.7327 |
| **CRP on POD3** | 150 | 0.7586 | 142 | 0.7394 |
| POD: post-operative day; CRP: C reactive protein, AUC: area under the curve | | | | |

**Suppl. Figure 1**

A – Receiver-operative characteristics (ROC) curve for C-reactive protein (CRP) on postoperative day 2 (POD2) for patients under 70 years.

B – Receiver-operative characteristics (ROC) curve for C-reactive protein (CRP) on postoperative day 2 (POD2) for patients over 70 years.

C – Receiver-operative characteristics (ROC) curve for C-reactive protein (CRP) on postoperative day 3 (POD3) for patients under 70 years.

D – Receiver-operative characteristics (ROC) curve for C-reactive protein (CRP) on postoperative day 3 (POD3) for patients over 70 years.

**Suppl. Figure 1.**


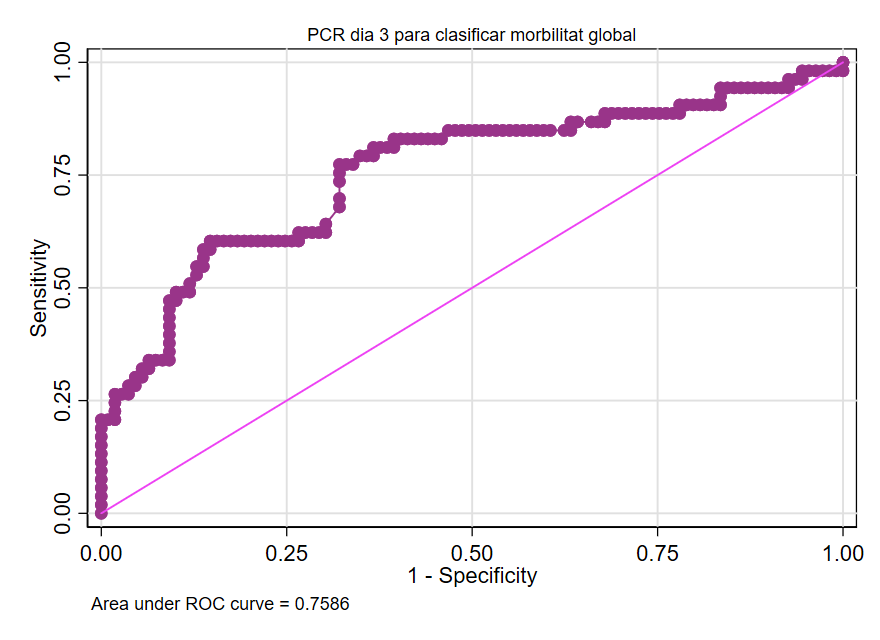


**A**

CRP on POD2 for < 70 years old patients

**B**


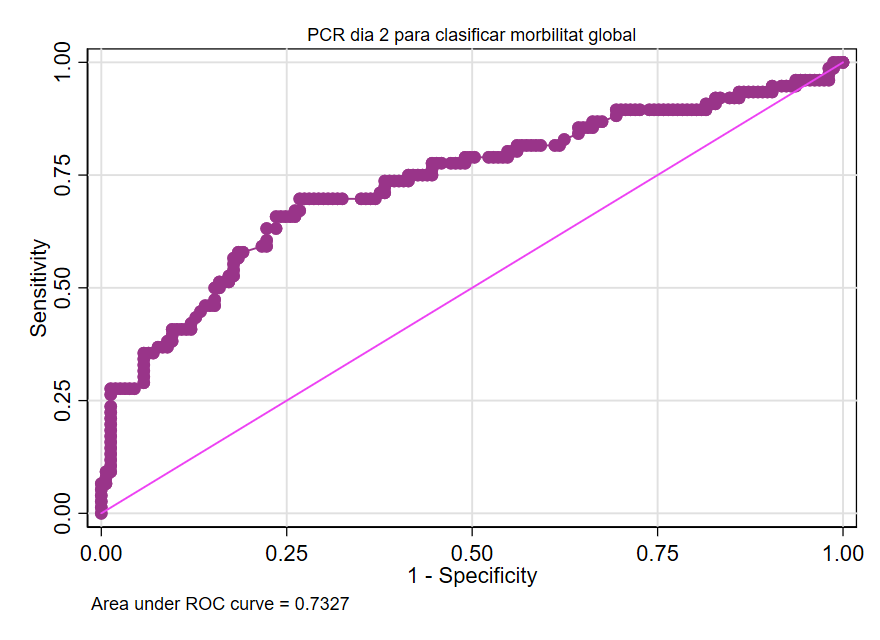


CRP on POD2 for > 70 years old patients

**C**


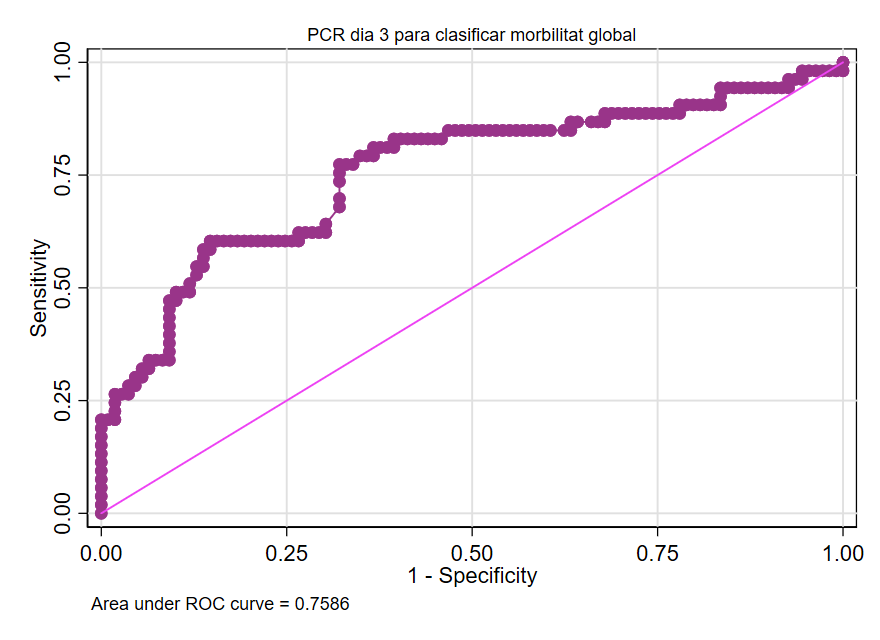


CRP on POD3 for < 70 years old patients

**D**


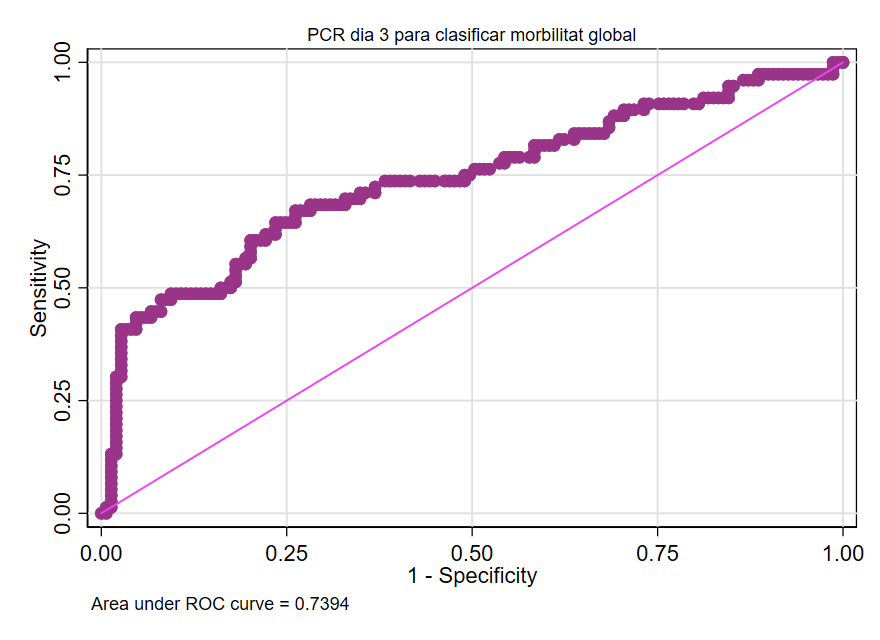


CRP on POD3 for > 70 years old patients

CRP on POD3

CRP on POD4
